# Estimating small-area population density in Sri Lanka using surveys and Geo-spatial data

**DOI:** 10.1371/journal.pone.0237063

**Published:** 2020-08-05

**Authors:** Ryan Engstrom, David Newhouse, Vidhya Soundararajan

**Affiliations:** 1 George Washington University, Washington, DC, United States of America; 2 The World Bank, Washington DC, United States of America; 3 Indian Institute of Management Bangalore, Bengaluru, India; International Food Policy Research Institute, UNITED STATES

## Abstract

Country-level census data are typically collected once every 10 years. However, conflicts, migration, urbanization, and natural disasters can rapidly shift local population patterns. This study demonstrates the feasibility of a “bottom-up”-method to estimate local population density in the between-census years by combining household surveys with contemporaneous geo-spatial data, including village-area and satellite imagery-based indicators. We apply this technique to the case of Sri Lanka using Poisson regression models based on variables selected using the Least Absolute Shrinkage and Selection Operator (LASSO). The model is estimated in villages sampled in the 2012/13 Household Income and Expenditure Survey, and is employed to obtain out-of-sample density estimates in the non-surveyed villages. These estimates approximate the census density accurately and are more precise than other bottom-up studies using similar geo-spatial data. While most open-source population products redistribute census population “top-down” from higher to lower spatial units using areal interpolation and dasymetric mapping techniques, these products become less accurate as the census itself ages. Our method circumvents the problem of the aging census by relying instead on more up-to-date household surveys. The collective evidence suggests that our method is cost effective in tracking local population density with greater frequency in the between-census years.

## Introduction

Up-to-date estimates of population density in small areas are valuable inputs for policymakers [1, 2]. They could, for example, facilitate efficient delivery of public goods and services and infrastructure projects [3]; track net migration patterns, especially in response to civilian conflicts, political upheavals, and climate tragedies; and help us better understand the impact of geographically-targeted economic policy interventions, such as Special Economic Zones. Traditional population data sources do not meet these requirements, as censuses provide local population measurements infrequently, typically decennially. Although household surveys can yield more frequent population estimates, they do not cover the entire country, and are not representative at small administrative levels, particularly in the developing world. The challenge of tracking population is exacerbated in low- and lower-middle- income countries where population growth rates are high and net migration patterns are rapid [4].

This study implements a “bottom-up” method to generate local estimates of population density and counts by combining household surveys with geo-spatial data [2]. In the areas covered under the survey, the method models population density obtained from surveys as a function of geo-spatial data. The model is then used to predict population density in the non-surveyed areas. By implementing this approach using periodically available surveys and up-to-date information from geo-spatial indicators, small area population estimates can be obtained regularly in the between-census years.

Commonly used techniques for small-area population estimation typically redistribute census population “top-down” from higher to lower administrative units using areal weighting approaches or dasymetric mapping techniques. Even though these methods are popular, and have continued to be refined by incorporating geo-spatial data and advanced statistical techniques, they are constrained by one major limitation: they rely heavily on the input-population data, that is, on the census [2, 5]. However, censuses may not always be available, are not conducted often, and may quickly become outdated due to frequent migration patterns, rapid and uneven population growth, or delays due to conflict and instability. For example, censuses are outdated in countries such as Lebanon and Somalia, and postponed or cancelled in some others such as Afghanistan, Madagascar and the Republic of Congo, due to conflicts or political instability [2]. Our method, instead of relying on the census, exploits the updated demographic information available from periodic household surveys, and the widespread coverage, and up-to-date and granular information offered by geo-spatial data.

The technique is applied in the context of Sri Lanka using the Household Income and Expenditure Survey (HIES), a nationally-representative household survey. The HIES is used in combination with satellite-imagery- based indicators of various types and image resolution categories, and other types of geo-coded ancillary data to estimate population density at the *Gram Niladhari* (GN) division, the lowest administrative level in Sri Lanka.

To motivate this approach, we begin by probing the ability of geo-spatial indicators to predict population density using the census data. Specifically, we ask how accurately do geo-spatial data predict out-of-sample GN-level census population density, and whether the accuracy depends on sectoral classifications and the resolution of the satellite imagery. Next, we implement the bottom-up approach using the HIES, and ask how accurate are out-of-sample estimates in comparison with the actual census density. Finally, we compare the accuracy of our estimates with the accuracy of other bottom up estimates in the literature, and other top-down estimates produced by open-source population products.

This study fits into a rapidly growing literature on estimating population in small areas. Top-down approaches remain popular. Open-source population products that use this approach include the Landscan, Facebook’s High Resolution Settlement Layer (HSRL), Gridded Population of the World (GPW), WorldPop, and Global Human Settlement Layer (GHSL). Additionally, studies have focused on redistributing population counts using a Random Forest model-based weighting scheme in Cambodia, Vietnam, and Kenya [1], in redistributing population density in Peru using satellite-imagery based covariates employing regression and tree-based methods [6], in downscaling population counts using one billion mobile phone call records from Portugal and France [5].

Relatively few studies use bottom-up techniques, and even fewer validate the accuracy of their estimates against the census. Earlier applications of these methods used coarse satellite imagery [7], focused on high-income countries where there are fewer data limitations (for instance, [8] in the Netherlands, and [9] in Australia), or validated the accuracy of their estimates only in larger administrative areas, but not in small-areas, and focused on for children under 5 years of age, and not the overall population [10].

In addition to the literature on population estimation, this study also speaks to the broader literature that uses satellite data, and statistical- and machine-learning- techniques to predict human welfare and demographic variables, and urban market boundaries [11–15].

We contribute to the literature by applying the bottom-up method in Sri Lanka, a lower-middle income country, where the census is conducted decennially, and where frequent small-area population density estimates would be a valuable policy input. To implement our approach, we compile satellite imagery based indicators of various resolutions from several open-source sources, and use texture and object features obtained from [13] that were in turn derived from very-high resolution imagery sourced from the company, Maxar (formally DigitalGlobe). We merge these satellite-based indicators, and other geo-spatial data, such as, area measures, with the HIES to create a unique dataset at the *Gram Niladhari* level.

Results indicate that the accuracy of our estimates are higher than prior bottom-up estimates using satellite data of similar dimension and resolution [8, 9], and most other population products using the top-down approach. Our results make a case for country statistics departments or ministries to utilize geo-spatial data and surveys to closely track population changes in their lower administrative divisions in a cost-effective manner.

## Materials and methods

**Fig 1:**
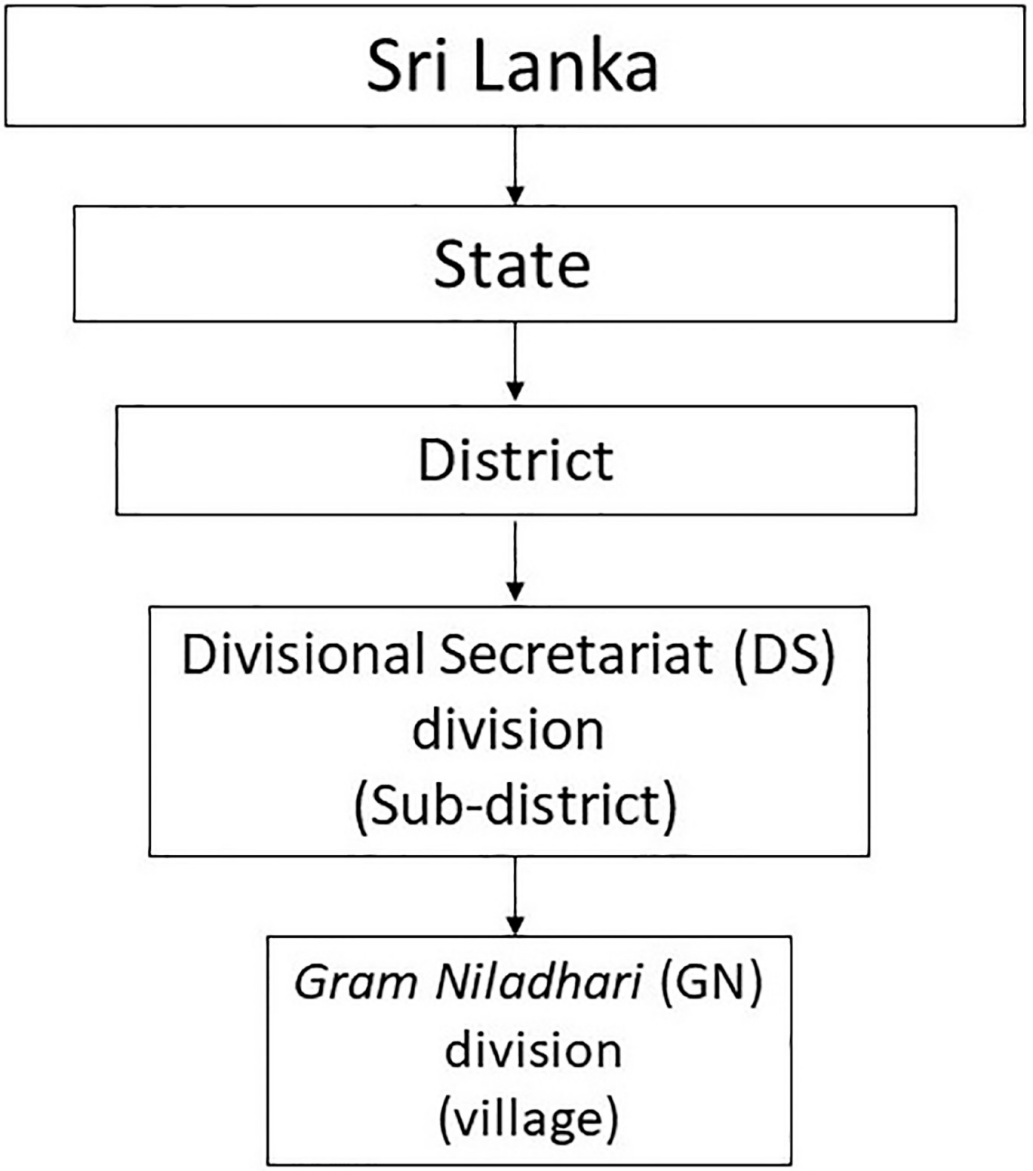
Hierarchical ordering of administrative divisions in Sri Lanka. Administrative divisions from the highest to the lowest level.

For better illustration of the details presented in this section, the hierarchical ordering of administrative divisions in Sri Lanka is presented in Fig 1. There are about 14,000 GN divisions that form the lowest administrative units. These divisions have an average area of 4.75 *km*^2^, and are similar in size to a village in many developing country settings, and we henceforth, for ease of exposition, refer to GN divisions as “villages”. While the term “village” commonly indicate settlements in rural areas in many countries, the usage in our context also includes small administrative units in urban areas. There are about 331 Divisional Secretariat divisions. These divisions are at one level higher than the villages, and one level lower than the districts, and henceforth are referred to as “sub-districts”.

### Population data sources

Our main sources of population density at the village level are the Census of Population and Housing, Sri Lanka, 2012, and the 2012-13 Sri Lankan Household Income and Expenditure Survey (HIES). The census universally covers all households in all of Sri Lanka’s 14, 000 villages. The HIES covers about 25,000 households in 2, 421 villages, spanning all the districts and sub-district divisions in the country. While the census is conducted decennially, the HIES is typically conducted once in three years.

The HIES follows a two-stage stratification process: the census blocks form the Primary Sampling Units (PSUs), and the households form the Secondary Sampling Units (SSUs) or Final Sampling Units (FSUs). The PSUs used in the HIES are census blocks, which are portions of villages. There were 62,571 PSUs versus 13,984 villages in the 2012 census. Therefore, the HIES can yield direct density estimates only at the PSU-level, and not at the village-level. Unfortunately, it is not possible to model population density at the PSU-level due to the lack of a PSU-level Geographical Information System (GIS) boundaries.

We therefore indirectly estimate the village population density using the HIES survey weights under the assumption that the survey weights reflect the inverse probability of the housing units being selected into the sample. This is approximately equivalent to scaling up estimated PSU population derived from the survey using that PSU’s share of the village population in the census. The steps used to estimate village level population density from the HIES are described in S1 Appendix. In order to apply this method, we limit the HIES sample to the 97 percent of villages that contain exactly one surveyed PSU in the village. The correlation in the HIES sample between the estimates of population density derived from the survey, and the actual village population density obtained from the census, is 0.91. This strong correlation indicates that this method, when applied in our context, generates credible village-level population density estimates.

### Geo-spatial data

The satellite imagery based geo-spatial data we use are categorized into four types, based on their resolution, availability, and the nature of the extracted data. Most indicators are obtained around 2012 (with a gap of +/- 2 years), the year around which the models are estimated. First, we use low-resolution open-source indicators from three sources: (1) Night time lights from the Visible Infrared Imaging Radiometer Suite (VIIRS) at a resolution of 750 m per pixel. We use the maximum and mean intensity of two months, namely, March and September, 2014; (2) Global Forest Change data based on [16], from which we use mean tree cover in 2000, and gain and loss in forest area between 2000 and 2014, at a resolution of 30 meters per pixel; (3) Advanced Spaceborne Thermal Emission and Reflection Radiometer (ASTER)’s elevation and slope data at a resolution of 30 meters per pixel in 2009. Although elevation and slope data are slightly older, these measures do not typically change over time, and hence it might be still appropriate to use them.

Second, we use open-source built-up area measures based on higher resolution imagery. The Global Urban Footprint (GUF) (year 2012) and Global Urban Footprint plus (GUF+) (year 2015) provide built-up estimates at a resolution of 12 meters per pixel. While GUF was constructed using the TerraSAR-X/TanDEM-X satellites that are based on radar-detection, GUF+ additionally incorporates data from the Sentinel and Landsat satellites that are based on optical remotely sensing. We also use Global Human Settlement Layer’s (GHSL) built-up estimates that are at approximately 38 meters per pixel and Facebook’s High Resolution Settlement Layer (HRSL) built-up area measure, available at a resolution of 30 meters per pixel.

Additionally, we use geo-spatial indicators extracted by [13] from very-high resolution commercially available satellite imagery acquired from the company, Maxar (formally DigitalGlobe). These indicators cover about 1,178 to 1,275 villages within 55 sub-districts in the country. The list of these 55 sub-districts is available in S2 Appendix. These imagery were mostly acquired for the years 2011 and 2012, although some were also captured for the year 2010. Our third set of indicators include texture features extracted by [13], including Fourier transform, Gabor filter, Histogram of Oriented Gradients (HoG), Line Support Regions (LSR), Pantex, Normalized Difference Vegetation Index (NDVI), and Speed-Up Robustness Features (SURF). Fourth, we use object-based features extracted by [13], including the number of cars, building count and size, roof type, shadow pixels, road length and type, and type of farm land (paddy or plantations). The features are identified using deep learning-based convolutional neural networks (CNN) and object based image processing software. More details on these features can be found in [13]. The data purchased and indicators derived by [13] costed $125,000 in the year 2015, but these data are considerably cheaper now.

The other geo-spatial variables we employ include the logarithm of village-area, and locational indicators such as binary indicator variables for the districts and the sector. There are 25 districts in Sri Lanka. The official sector classification includes the urban, rural, and estate sectors. Although 91% of the villages have a unique sectoral assignment, there are multiple sectors associated with each village in the remaining 9%. To simplify, we first create the following modified sectoral definition: (1) rural or estate; (2) urban, and then assign villages with multiple assignments to the urban sector.

Table 1 concisely presents the key variables used in the study, along with their source, time frame, resolution type, and geographical coverage. Note that all data sources are Tables 2 and 3 present the summary statistics for the population variables and geo-spatial indicators for the national sample and the 55 sub-districts, respectively.

**Table 1:** Satellite indicators by resolution and availability.

Indicator	Source	Time-frame	Coverage	Resolution	Availability
*A. Population*					
Population	Census		National		Open-source
Population	HIES		1421 villages		Open-source
*B. Geo-Spatial*				
Night time lights	VIIRS	2014	National	Low	Open-source
Elevation	ASTER	2009	National	Low	Open-source
Slope	ASTER	2009	National	Low	Open-source
Tree cover	[16]	2000	National	Low	Open-source
Tree cover gain/loss	[16]	2000 to 2014	National	Low	Open-source
Built-up area	GUF, GUF+	2012, 2015	National	High	Open-source
Built-up area	GHSL	2014	National	High	Open-source
Built-up area	Facebook	2015	National	High	Open-source
Built-up area	[13]	2011, 2012	55 sub-dist.	High	Commercially-available
Cars	[13]	2011, 2012	55 sub-dist.	High	Commercially-available
Shadows	[13]	2011, 2012	55 sub-dist.	High	Commercially-available
Roof type	[13]	2011, 2012	55 sub-dist.	High	Commercially-available
Road type	[13]	2011, 2012	55 sub-dist.	High	Commercially-available
Agricultural land type	[13]	2011, 2012	55 sub-dist.	High	Commercially-available
Paddy land type	[13]	2011, 2012	55 sub-dist.	High	Commercially-available
NDVI	[13]	2011, 2012	55 sub-dist.	High	Commercially-available
Other Texture Ind.	[13]	2011, 2012	55 sub-dist.	High	Commercially-available

HIES = the Household Income and Expenditure Survey; VIIRS = Visible Infrared Imaging Radiometer Suite; ASTER = Advanced Spaceborne Thermal Emission and Reflection Radiometer; GUF = Global Urban Footprint; GHSL = Global Human Settlement Layer; NDVI = Normalized Difference Vegetation Index. [13] use commercially-available imagery from Maxar (formally DigitalGlobe) to derive features and textures for 55 sub-district divisions. “Other Texture indicators” include SURF, Pantex, and Histogram of Oriented Gradients. The time frame refers to the year(s) during which the satellite images were obtained. While NDVI data are generally public, the NDVI values in Engstrom et al. 2017 were we calculated on the commercial imagery. Hence it is listed under commercial rather than open source data.

**Table 2:** Summary statistics for villages in the national sample.

Indicator	Mean	Std. Dev.	Min	Max
*Geographic Descriptors*				
Log village area	1.25	0.81	0.04	6.35
Urban indicator	.092	0.28	0.00	1.00
*Population Summary*				
Village Census Population Density(per km^2^)	1,400	2,703	0	50,126
Village HIES Population Density(per km^2^; N = 2,348)	1,996	3,394	0.607	49,209
*Low Resolution Open-source Indicators*				
Night-time lights March 2014—Mean	0.98	2.25	0.000	72.57
Night-time lights March 2014—Maximum	1.45	4.13	0.000	274.93
Night-time lights September 2014—Mean	0.95	2.04	0.000	56.61
Night-time lights September 2014—Maximum	1.39	3.28	0.0	170.62
Mean Elevation	197.13	313.35	3.4	2,214.55
Mean Slope	8.68	5.15	1.2	28.79
Mean Tree Cover	46.93	26.91	0.0	97.26
Gain in Tree Cover	0.02	0.02	0.000	0.25
Loss in Tree Cover	0.005	0.012	0.000	0.330
*High Resolution Open-source Indicators*				
GUF Built-up Area	10.60	20.37	0.0	100.00
GUF+ Built-up Area	14.21	25.83	0.0	101.05
GHSL Built-up Area	15.41	24.88	0.0	100.00
Facebook Built-up Area (N = 13,437)	15.65	19.16	0.0	99.55
Observations (N)	13,970			

HIES = Household Income and Expenditure Survey; GUF = Global Urban Footprint; GHSL = Global Human Settlement Layer; NDVI = Normalized Difference Vegetation Index.

**Table 3:** Summary statistics for villages in the 55 sub-districts sample.

	Mean	Std. Dev.	Min	Max
*Geographic Descriptors*				
Log village area	0.97	0.60	0.09	4.27
Urban indicator	0.28	0.45	0.00	1.00
*Population Summary*				
Village census population density	2621	3507	22	43,984
Village HIES population density(per km^2^; N = 414)	2,821	3,576	10.22	38,609
*Low Resolution Open-source Indicators*				
Night-time lights March 2014—Mean	1.39	3.27	0.00	57.21
Night-time lights March 2014—Maximum	2.10	8.54	0.00	274.93
Night-time lights September 2014—Mean	1.26	2.50	0.00	28.96
Night-time lights September 2014—Maximum	1.76	3.88	0.00	92.13
Mean Elevation	209.96	428.52	5.56	2214.55
Mean Slope	7.63	5.14	1.15	23.89
Mean Tree Cover	44.22	26.35	0.00	92.24
Gain in Tree Cover	0.02	0.03	0.00	0.22
Loss in Tree Cover	0.01	0.02	0.00	0.33
*High Resolution Open-source Indicators*				
GUF Built-up Area	21.81	26.92	0.00	97.11
GUF+ Built-up Area	31.68	36.52	0.00	100.11
GHSL Built-up Area	33.59	35.68	0.00	100.00
Facebook Built-up Area (N = 1,275)	30.16	28.18	0.67	97.43
*Indicators Calculated from Commercial, Very High Resolution Imagery Road variables*				
% of roads that are minor paved (4 m width)	6.78	15.50	0.00	100
% of roads that are main paved (5 m width)	11.32	15.07	0.00	100
% of roads that are paved city (4 m width)	11.74	17.64	0.00	92.76
*Building Density and Vegetation*				
% shadow pixels covering valid area (N = 1,353)	5.80	5.47	0.27	38.17
NDVI, mean scale 32	0.52	0.17	0.00	0.95
Total built-up area	69,757	68,699	0	7,08,867
*Roof type*				
Fraction of total roofs that are clay	35.80	20.67	0.00	100
Fraction of total roofs that are aluminum	14.26	7.27	0.00	71.92
Fraction of total roofs are asbestos	7.79	11.66	0.00	71.20
*Cars*				
log number of cars (N = 1,252)	3.42	1.02	0.88	8.30
*Agricultural Land*				
% of Village agriculture that is paddy	44.46	37.65	0.00	100.00
% of Village agriculture that is plantation	55.14	37.58	0.00	100.00
*Textural and spectral characteristics*				
Pantex (human settlements) mean, scale 8m	0.56	0.50	0.00	3.95
Pantex (human settlements) mean, scale 32m	0.66	0.59	0.00	4.68
Gabor filter (scale 64m, features 6), mean	0.67	0.32	0.01	2.03
Gabor filter (scale 64m, features 14), mean	0.68	0.31	0.02	1.95
Histogram of Oriented Gradients (scale 16m), mean	37.91	9.66	0.00	146.18
Observations (N)	1,360			

HIES = Household Income and Expenditure Survey; GUF = Global Urban Footprint; GHSL = Global Human Settlement Layer; NDVI = Normalized Difference Vegetation Index. The NDVI scale refers to the size of the window used to calculate average NDVI. Summary statistics of LASSO-selected indicators across all models are reported.

### Modeling

We use Poisson regressions to model population density at the village level. This takes the following form:

$$\begin{array}{c} log\left(E\left(P_{v}|X_{v},Z_{v}\right)\right)=\alpha +\beta X_{v}+\gamma Z_{v}, \end{array}$$ (1)

where *P*_*v*_ is the population density (persons/km^2^) of village *v*. *X*_*v*_ is a vector of satellite imagery-based indicators defined for village *v*. *Z*_*v*_ is a vector that includes the natural logarithm of village area (*Ln area*_*v*_), an indicator variable for urban areas, and indicator variables for Sri Lanka’s districts.

To prevent model over-fitting, we employ the least absolute shrinkage and selection operator (LASSO) regularization which estimates a regression model with an added constraint that enforces parsimony. We follow a two-step procedure. First, a full set of variables is included, on which LASSO regularization is conducted to choose variables. Second, the final Poisson model is estimated using the chosen variables. Eq 2 presents the objective function for LASSO regularization. Let *τ* represent the vector of all coefficients, *β* and *γ*, put together, of size *p*.

$$\begin{array}{c} \underset{\alpha ,\tau }{\text{min}}\;-\frac{1}{N}l\left(\alpha ,\tau |X_{v},Z,P_{v}\right)+\lambda \sum_{j=1}^{p}\left|\tau_{j}\right|, \end{array}$$ (2)

where *l* is the value of the log likelihood function of the Poisson model using parameters *α* and *τ*, and λ is a non-negative regularization parameter. While setting λ = 0 yields unconstrained Poisson regression estimates, a large λ penalizes the absolute values of the elements of *τ*. Fivefold cross validation is applied to choose the value of λ that minimizes the root-mean squared error (RMSE) across the folds. The in-sample *R*^2^ from this step indicates the goodness of the model fit. Since LASSO also generally shrinks the magnitude of all coefficients towards zero [17], we avoid biased predictors by running a simple Poisson model based on the Lasso selected variables. From this, we obtain the out-of-sample *R*^2^, mean absolute error (MAE), and RMSE, all of which indicate out-of-sample predictive accuracy. For estimations, we use the glmnet package in *R* which we invoke from Stata, using the wrapper, *rlasso* [18].

First, we implement the above model using the village level census population density as the dependent variable (in the census year, 2012) to probe how well geo-spatial data can predict population density. We conduct this exercise for a model in the entire country separately using different indicators: (i) district and urban-area binary indicators; (ii) including log village-area to i; (iii) including low-resolution open-source indicators to ii; (iv) including higher resolution open-source indicators to iii. Doing this enables us to compare prediction accuracy between higher versus lower resolution data in the national sample. In the 55 sub-districts where the features from [13] are available, we implement models using: (v) texture-features plus indicators in iv; and (vi) object-features plus the indicators used in v. For the 55 sub-districts, we compare prediction accuracy successively from models in (i) through (vi). We also repeat the analysis separately for urban areas, and for rural/estate areas.

We conduct two robustness checks. Focusing on the 55 sub-districts, we reduce the training sample size to one-half and to one-quarter of the original sample, to examine if the prediction accuracy is sensitive to the size of the training sample. We also implement a flexible random forest algorithm (using the randomForest package in *R*) using population density as the response variable, and employing the different sets of geo-spatial data described above, and report out-of-sample accuracy measures.

Next, we implement the bottom-up method focusing on the 1,178 villages in the 55 sub-districts sub-sample for which commercially-available texture and object features were obtained. Out of these villages, the HIES was conducted in 414 villages. In this sub-sample, we estimate Eq 1 using the HIES density as a function of all the geo-spatial indicators, including the commercially-procured satellite indicators. Note that the dependent variable, population density, is now measured for the year 2012-2013, the year the HIES was conducted. Since this is a survey-based model, we use the inverse of village population for village *v* as weights ($\frac{1}{pop_{v}}$). In addition, there could be factors correlated with geo-spatial indicators that affect the probability of selection of the village into the HIES. We also, therefore, adjust the weights based on the predicted probability of the village being sampled into the HIES based on geo-spatial indicators available for all villages [19, 20]. This probability is obtained from the following probit model:

$$\begin{array}{c} IN_{v}=\eta_{0}+\eta_{1}\;lasso_{v}+\epsilon_{v}, \end{array}$$ (3)

where *IN*_*v*_ is a binary indicator for whether village *v* is sampled into HIES, and *lasso*_*v*_ represents all LASSO-selected variables from the Poisson model. We estimate Eq 3 using data for all the 1,178 villages in the 55 sub-districts. The corrected weights based on the probability of the village selection into the HIES, $I\hat{N_{v}}$, is given by $\frac{1}{\left(pop_{v}\times I\hat{N_{v}}\right)}$.

Using the estimated model, we predict out-of-sample densities for the remaining 946 non-HIES villages and report out-of-sample accuracy measures by comparing them with the actual census density. To provide additional context, we compare the accuracy of this model with the accuracy of estimates from a similar model that uses census-density as the dependent variable, and with the accuracy of the estimates of other bottom-up estimates in the literature, and with the accuracy of open-source top-down population products. Further, we calculate population count estimates by multiplying these density estimates with village-level area, and report their accuracy with respect to the census population.

## Results

### Predictive power of Geo-spatial data

Poisson regression results using the census data based on the variables selected from LASSO regularization indicate that geo-spatial indicators have strong predictive power in predicting village level population density (Table 4). The results are presented in panel A. At both the national level and in the 55-sub-districts sample, simple locational indicators do not explain much of the variation in population density. The out-of-sample estimation *R*^2^ is only about 0.36. However, adding log village area as an explanatory variable tremendously improves the out-of-sample *R*^2^ to 0.65 in the national sample and to 0.589 in the 55-sub-districts. At the national level, conditional on the locational indicators and village size, the value added in using low-resolution open-source indicators (out-of-sample *R*^2^ is 0.702), and additionally using high resolution open-source indicators (out-of-sample *R*^2^ is 0.75) is moderate but non-trivial. In the 55 sub-districts, adding commercially-procured texture features to open-source imagery-based models does not improve the prediction accuracy (out-of-sample *R*^2^ remains at around 0.7). However, adding object features to the model improves the out-of-sample *R*^2^ by 10 points (0.83). For the interested reader, the marginal effects of the full set of LASSO-selected variables from the model using all geo-spatial indicators are provided in S1 Table.

**Table 4:** Accuracy of population density estimates.

Geo-spatial Indicators						Accuracy Measures		No. of variables		Villages
Loc. dummy	Village area	Low-res (open)	High-res (open)	Texture (comm.)	Object (comm.)	In-sample *R* ^2^	Out-of-sample *R* ^2^	Candidate	Selected	
A. All sectors										
i. National Sample										
×						0.499	0.359	25	22	13,970
×	×					0.806	0.650	26	15	13,970
×	×	×				0.843	0.702	35	16	13,970
×	×	×	×			0.888	0.750	39	11	13,437
ii. 55 sub-districts										
×						0.422	0.361	25	5	1,428
×	×					0.731	0.589	26	6	1,428
×	×	×				0.804	0.677	35	8	1,428
×	×	×	×			0.858	0.710	39	7	1,428
×	×	×	×	×		0.874	0.755	46	9	1,343
×	×	×	×	×	×	0.918	0.830	67	21	1,178
B. Rural/Estate										
i. National Sample										
×						0.256	0.146	25	22	12,686
×	×					0.757	0.646	26	23	12,686
×	×	×				0.806	0.726	35	24	12,686
×	×	×	×			0.877	0.808	39	19	12,389
ii. 55 sub-districts										
×						0.217	0.179	25	6	1,046
×	×					0.752	0.688	26	7	1,046
×	×	×				0.815	0.754	35	10	1,046
×	×	×	×			0.898	0.838	39	4	935
×	×	×	×	×		0.919	0.882	46	26	935
×	×	×	×	×	×	0.942	0.869	67	33	881
C. Urban										
i. National Sample										
×						0.243	0.159	25	4	1,284
×	×					0.595	0.493	26	2	1,284
×	×	×				0.654	0.540	35	5	1,284
×	×	×	×			0.690	0.569	39	6	1,048
ii. 55 sub-districts										
×						0.189	0.160	25	1	382
×	×					0.487	0.413	26	2	382
×	×	×				0.637	0.511	35	5	382
×	×	×	×			0.665	0.525	39	5	340
×	×	×	×	×		0.740	0.668	46	6	340
×	×	×	×	×	×	0.860	0.804	67	31	336

Open indicates open-source indicators, and comm. refers to commercially-procured indicators. The results are based on a Poisson regression model whose dependent variable is the census village population density, and whose independent variables are selected based on LASSO regularization in the respective models using different sets of variables: “Loc. dummy” indicates locational indicators, namely, district fixed effects and an indicator for urban villages. “Village area” indicates log village-area. “Low-res (open)” and “High-res (open)” indicators refer to the low-resolution and high-resolution open-source indicators respectively, as defined in Table 1. “Texture (comm.)” and “Object (comm.)” indicators refer to very high-resolution texture and object features respectively from [13] as defined in Table 1. The out-of sample *R*^2^ is obtained from stata’s crossfold command using five-fold cross-validation.

The results separately for the rural/estate, and urban sectors are presented in panels B and C respectively in Table 4. Log village area remains a strong predictor of population density separately in both sectors. Using the full national sample, the open-source satellite indicators explain more variation in population density in the rural/estate sectors (0.808) than the urban sector (0.569). In the sample with 55 sub-districts, the predictive power of urban population density increases tremendously when adding the commercially-procured texture and object features, relative to only including open-source indicators (0.804 compared to 0.525). Adding commercially-procured indicators leads only to a limited improvement in prediction accuracy in rural areas (0.869 compared to 0.838). This result is consistent with greater uniformity in the relationship between built-up area and population density in rural areas, where simple measures of built-up area are sufficient to predict population density. In urban areas, due to the complex nature of the relationship between population density and buildings, where the latter can be both commercial and residential, advanced measures such as object classifiers and texture measures are required for better prediction.

As a robustness check, we show that Random forest (RF) models provide similar results in terms of out-of-bag *R*^2^, RMSE, and MAE, overall, and across resolution-types and sector-types (S2 Table). These results collectively indicate that LASSO selection performs as well as random forest models in this setting. While random forest models are known to outperform LASSO models in some contexts [21], these conclusions are based on linear models. Our usage of Poisson regressions may negate the predictive advantage of random forest models, which are traditionally better able to account for non-linear relationships. Further, studies predicting population counts and density have specifically shown mixed results on comparative performances between random forest and LASSO-based estimates [6].

We also show that the accuracy of our estimates is robust to changing the size of the training data (S3 Table). Using a sample of 1,178 villages in the 55 sub-districts, the out-of-sample *R*^2^ increases marginally to 0.843 if we reduce the training sample by half, with a mild reduction to 0.832 if we further reduce the sample size by another one-half. There is only a small increase in the MAE and RMSE with the reduced sample sizes.

### The bottom-up method using survey and Geo-spatial data

While it is encouraging to see that geo-spatial data accurately predict census population density at the village level, these results do not show that surveys combined with geo-spatial data can be used to approximate population density. We now turn to examining this by testing the accuracy of a survey-based model in the 55 sub-districts sample. We estimate a Poisson model of HIES population density in the HIES-villages, and then obtain out-of-sample density estimates in the non-HIES villages.

The results from a model using all geo-spatial indicators, including open-source and commercially-procured imagery-based indicators are provided in panel A in Table 5. For the model using the inverse of village population as weights, the *R*^2^, Spearman Rank Correlation (SRC), and the MAE between the density estimates and the census density are 0.79, 0.91, and 664, respectively (columns 1 to 3). If the weights are corrected based on the probability of the village being selected into the HIES, we obtain similar results: 0.78, 0.92, and 665 for the same measures. In panel B, we present the same results based on a model using only open-source satellite imagery. The out-of-sample prediction accuracies are very similar in this case (0.75, 0.92, and 663, and 0.77, 0.92, and 657 for the three measures using the two types of weights). From both panels A and B, we can see that the HIES-based model performs equally well compared to a similar model that uses census-density as the dependent variable.

**Table 5:** Out-of-sample accuracy of HIES-based village population density estimates using open-source and commercially-procured satellite data, 55 sub-districts.

	Population Density			Population Count				
	*R*^2^	SRC	Mean AE	*R*^2^	SRC	Mean AE	Mean RE	Median RE
	(1)	(2)	(3)	(4)	(5)	(6)	(7)	(8)
*A.Model estimates, open-source & commercially-procured satellite imagery*								
HIES-based	0.79	0.91	664	0.60	0.67	607	37%	28%
HIES-based (balance correction)	0.78	0.92	665	0.58	0.67	607	35%	29%
Census-based	0.81	0.91	679	0.60	0.65	644	50%	28%
*B.Model estimates, open-source satellite imagery*								
HIES-based	0.75	0.92	663	0.56	0.69	595	34%	28%
HIES-based (balance correction)	0.77	0.92	657	0.55	0.69	606	34%	28%
Census-based	0.76	0.91	750	0.56	0.63	739	58%	36%

HIES = the Household Income and Expenditure Survey; SRC = Spearman Rank Correlation; AE = Absolute error; RE = Relative Error (with respect to the census). We retain only the 1,178 villages in the 55 sub-districts for which the commercially-procured data are available. The model is estimated using population density as the dependent variable on sub-sample of 414 villages covered in 2012-2013 HIES survey. All models include district fixed effects, urban-area dummy, and log village area. Out-of-sample prediction accuracy (with the census) are conducted on the 946 villages not covered in the HIES. The weights for the HIES-based model are $\frac{1}{Pop_{v}}$. The balanced corrected weights are $\frac{1}{\left(Pop_{v}\;XI\hat{N_{v}}\right)}$. Population count estimates were obtained by multiplying density estimates with village-area. The accuracy parameters for population estimates are based on comparisons with the actual census population. The mean and median RE are mathematically the same for population density and population count estimates.

We multiplied these density estimates with village-level area to obtain population count estimates. The accuracy measures based on population counts are reported in columns (4)-(6). The *R*^2^ and SRC are generally lower for population counts compared to density estimates, but the MAEs are also lower. Further, we estimated a similar model using population count as the dependent variable, from which we directly obtained population estimates (S4 Table). Population count estimates derived indirectly from a density-based model are more accurate than those obtained directly from a population-count-based model.

The mean and the median relative errors are reported in columns 7 and 8, respectively. The relative errors are the same for population density and count estimates because the only difference between the two measures is the multiplicative factor, village-area. Using all geo-spatial indicators, the mean and median errors are 37% and 28%, and 35% and 29%, without and with the additional weight correction. Using only open-source imagery-based indicators, the mean and median REs are 34% and 28% for models without and with weights correction.

## Discussion

### Commercially-available versus open-source imagery

Table 5 predictionaccuracy provides the model accuracy using both open-source indicators and commercially-available indicators for the 55-sub district sub-sample in panel-A, and using the open-source imagery based indicators for the national sample (all sub-districts) in panel-B. It is striking that the accuracy of the model involving open-source imagery is marginally better than the accuracy of the model including the commercial-indicators. Therefore, while commercial indicators may be expensive, unavailable, or unobtainable in some instances in specific countries, our model without necessarily relying on them, works almost equally well with open-source imagery based indicators.

We estimated the model by rural and urban areas separately. These results are not reported in the paper, but are available upon request. Mirroring Table 5, in rural areas, the predictive power of the models do not change with and without high-resolution commercial imagery. However, in urban areas, where there is a complex nature of the relationship between population density and buildings as elaborated earlier, commercial imagery does increase the predictive power much more compared to a model that only has low and medium resolution open-source imagery.

### Cost effectiveness

The data purchased and indicators derived by [13] cost $125,000 in the year 2015, although these data are considerably cheaper now. Procuring satellite imagery to update population density in the between-census years would be considerably cheaper than repeating a whole census at frequent intervals. To provide a sense of how expensive a whole census is, we draw upon a study that examined the cost of conducting Ghana’s housing census [22]. Comparing with Ghana, if anything, underestimates the per person cost of census data collection in Sri Lanka because Ghana is comparatively less developed. The study estimates the cost of conducting the census in Ghana at $2.5/person. Given Sri Lanka’s total population of 20 million, the total cost of conducting the census in Sri Lanka works out at least $50 million dollars.

We make this cost comparison with the census not to indicate that our method can replace the decennial censuses, but to indicate that it cheaper to update population density estimates in the between-census years using our method, rather than to repeat full censuses more frequently. Furthermore, if we used only open-source imagery, for which the accuracy of the model estimates are similar, the modelling costs are even lower.

### Regular updating of satellite imagery

As much as the survey, the model relies crucially on the availability of updated geo-spatial data. It might be worthwhile to inspect if these data and indicators are available in regular intervals. Remarkably, many of the data sets in Table 1 are being regularly updated. For instance, the GHSL and the tree cover data are being updated, and GUF data has been updated with the release of the World Settlement Footprint which is in the process of being updated again now [23–25]. Further, all the commercial data used in this study can all be updated with new imagery as it is collected. While one of the open-source datasets, the Facebook data, may not be scheduled to be updated regularly, remarkably, the prediction accuracy does not fall if we drop Facebook built-up area from the model (these results are not presented in the paper, but available upon request).

Moreover, the costs for acquiring these data are dropping as more companies are able to produce the data and more satellite providers collect the data. Possibly better data sets will be created in the future that could be used in similar approaches described in this manuscript. The Gates Foundation is funding building-footprint extraction for most of Africa from very high spatial resolution imagery and plans to provide the data to research such as this [26]. We have seen some output from this work, and used them in other studies and found them to be very accurate; these data would be useful for modeling such as the present study. Additionally, similar work is being done by Microsoft in the US, that has also released these data to the public. Some of us have been in discussions with them in expanding this to other countries. Other sources of data, such as the Open Street Map are getting better and more complete every day [27]. Some of the inputs used such as slope and elevation, while important for modeling, do not change over time. Taken together, while most of the data sets used in this study are being regularly updated, there will be plenty of data (and possibly better) to do this type of work as time progresses.

### Comparison with other bottom-up estimates

The out-of-sample *R*^2^s for our bottom-up estimates range from 0.75 to 0.79 (Table 5). This is higher than the out-of-sample *R*^2^ of 0.739 (the square of the reported correlation coefficient of 0.86) between original and estimated population density obtained for the case of Australia at the level of the Australian census collection districts [9]. Our median RE at 28% is lower than those reported for the Netherlands using geo-spatial data of similar dimension and resolution as ours, which ranges from 42% to 85.4% [8]. These higher errors could be attributed to the study’s usage of fewer satellite indicators, linear rather than non-linear regressions, and simple rather than stratified random sampling in the survey. Although [8] report lower errors (18.3% or 18.5%) by utilizing floor-space or volumetric (3-Dimensional) satellite data and functional information about buildings, these data are costly to obtain periodically in low-income countries.

### Comparison with open-source top-down estimates

We compare our estimates with the following five “top-down” population products: (1) Landscan for the year 2010; (2) WorldPop for 2010 and 2015; (3) Facebook HRSL for 2015; (4) Gridded Population of the World v3 (year 2010) and v4 (year 2015), and (5) GHSL for 2015. All sources employ a top-down approach using a combination of areal interpolation techniques, including basic dasymetric approaches in conjunction with ancillary data, and statistical modeling methods to distribute census population to smaller grids. While GPW redistributes data based on an equal weighting technique, other sources use measures such as built-up area, road count and density, elevation, slope, and light intensity (“covariates”) to proportionally redistribute population. Facebook, WorldPop 2015, GPW 2015, and GHSL estimates are based on the 2012 Sri Lankan census; WorldPop 2010 and GPW 2010 are based on the 2001 census; and Landscan uses mid-year population of the country in the past year calculated by the Geographic Studies Branch, US Bureau of Census [28]. Detailed information on the input population, ancillary data, and the redistribution methodology for each source is presented in S5 Table.

At first glance, it may seem odd that we use built-up area measures from Facebook and GHSL, and compare our population density estimates with the same sources. It is important here to distinguish between estimates of built-up area, top-down estimates of population density, and bottom-up estimates of population density. Currently, built-up area estimates are publicly provided by many companies including the Global Human Settlement Layer and Facebook. These estimates of built-up area can be produced regularly solely from satellite imagery, and do not require a ground-truth measure of population density. Our model uses these built-up area as explanatory variables to predict population density. These companies also independently combine their built-up area measures with census data to estimate local population density. They utilize top down methods that redistribute census population counts according to built-up area, and therefore become stale over time. In contrast, our method is a “bottom-up” method that calibrates the model to survey data and therefore can be implemented in between census years. The idea behind Table 6 is to compare our estimates with other top-down estimates in a census year.

**Table 6:** Accuracy of existing top-down population products.

	Population Density			Population Count				
	*R*^2^	SRC	Mean AE	*R*^2^	SRC	Mean AE	Mean RE	Median RE
	(1)	(2)	(3)	(4)	(5)	(6)	(7)	(8)
WorldPop 2015	0.99	1.00	312	0.99	0.99	237	13%	13%
Facebook HSRL 2015	0.91	0.97	417	0.90	0.93	309	20%	14%
GHSL Grid 2014	0.84	0.88	639	0.74	0.79	556	39%	26%
GPW 2015	0.68	0.90	1029	0.50	0.78	740	44%	27%
GPW 2010	0.29	0.28	1550	0.39	0.48	911	52%	35%
WorldPop 2010	0.31	0.81	1206	0.06	0.44	987	64%	34%
LandScan 2010	0.04	0.10	2562	0.01	-0.24	2012	135%	92%

HRSL = High Resolution Settlement Layer; GHSL = Global Human Settlement Layer; GPW = Gridded Population of the World; SRC = Spearman Rank Correlation; AE = Absolute error; RE = Relative Error (with respect to the census). We retain only the 1,178 villages in the 55 sub-districts for which the commercially-procured data are available. To be consistent with Table 5, the prediction accuracy with the census are reported for the 946 villages not covered in the HIES. The mean and median RE are mathematically the same for population density and population count estimates.

Using *R*^2^, SRC, and mean REs as metrics, our model estimates are better than all top-down estimates except Facebook and Worldpop 2015 (Table 6). Our median REs are higher than only Facebook and Worldpop 2015, and comparable to GHSL and GPW 2015, and much lower compared to GPW, Worldpop, and Landscan (all in 2010). The relative performance of our population count estimates in Table 5, in comparison to the top-down population estimates in Table 6 is similar to that of the population density estimates.

Table 6 accuracy shows that the top-down products using the most recent census for redistribution, and using higher resolution imagery-based covariates are performing better than the others. For example, GPW and WorldPop 2010 estimates use the 2001 census for redistribution, and hence it is not surprising that they perform poorly in comparison to the 2012 census. Similarly, an earlier unpublished version of Facebook’s estimates we worked with only yielded a correlation coefficient of 0.5 because Facebook initially used the 2001 Sri Lankan census for calibration. The lower accuracy of products that use the older census might seem intuitive. By the same argument, it is important to foresee that even the best performing products seen in Table 6 may become inaccurate with the passage of time because their source data, the census, eventually becomes outdated. In other words, while the product that uses the 2012 census for redistribution could estimate population density accurately in 2012, it might not predict density as accurately in the later years, until the next census is available (likely in 2022) and utilized. This reinforces our motivation for using bottom-up methods that combine up-to-date and periodic surveys with geo-spatial data to estimate population density, without having to rely on the census that is only available at most decennially in most countries, including Sri Lanka.

### Coefficient of variation of the estimates

We calculated the coefficient of variation (CV) of predicted density estimates for each sub-district based on the bottom-up method, and compared them with the full-survey based density estimates for the entire country. The CV from the predicted density values is based on the average of a Poisson model, with errors clustered at the sub-district level. It was calculated using the Poisson command in Stata followed by the margins, pred(n) noesample vce(unconditional) command. The average CV of population density calculated directly from the full survey, across 329 sub-districts, is 50.0. This is calculated using the Horvitz-Thompson approximation, which allows for positive correlation in population density across GN Divisions within sub-districts. The average CV of population density calculated from the model predictions, for the same 329 sub-districts, is 6.7. Therefore, in the full sample, moving from the survey to the model-based predictions generates an approximate 85 percent reduction in the size of the CV of sub-district population density.

## Conclusion

Many existing estimates of estimating local population use a top-down dasymetric mapping approach to distribute census data based on a set of covariates derived from satellite imagery and other ancillary data. These estimates are only as accurate as the source data—the census—and hence their accuracy declines as the census itself ages. We propose a bottom-up technique by pairing survey data with geo-spatial indicators using Poisson regressions employing variables selected based on LASSO regularization. This model can yield population density estimates in areas where the survey was not conducted, and can be repeated frequently using periodic survey and satellite data to frequently track population changes. We apply the method in the context of Sri Lanka to predict population density at the lowest administrative level, the Gram Niladhari (GN) division (village-level). Our model uses the Sri Lankan Household Income and Expenditure Survey (HIES), and predicts density out-of-sample in the non-HIES villages.

Four aspects of these results are particularly noteworthy. First, our performance is better than the two other studies that report out-of-sample validation of bottom-up population estimations using similar satellite data. Second, although our estimates are not as accurate as the most accurate top-down estimates, the model gives comparable or better performance than most others. However, because surveys are collected much more frequently than censuses, bottom-up methods that combine geo-spatial data with surveys have the crucial advantage of remaining up to date even if the census ages, unlike even the best performing top-down estimates. Third, the survey used to calibrate the model covers only 17% of the villages in Sri Lanka. The fact that the combination of geo-spatial data with this type of small survey estimates population density as accurately as the estimates that use an entire census demonstrates the cost-effectiveness of this approach. Fourth, overall, the predictive power of the models does not change with and without high-resolution commercial imagery. Simply using open-source low and medium resolution imagery would suffice to achieve adequate accuracy. However, in urban areas, as expected, high resolution- commercial imagery increase the predictive power much more compared to a model that only has low and medium resolution open-source imagery.

The main reason why this method is useful is because surveys are more frequent and less expensive than censuses, and geo-spatial data can be acquired routinely and with complete spatial coverage, and hence can potentially be more efficiently utilized to track population changes in small areas. It is important to clarify that even though the method does not directly rely on the census, it does not reduce the importance of the censuses which remain essential to provide the sampling frame for subsequent and periodic surveys. Moreover, the census data provide authoritative data in the years the censuses are conducted. In the between-census years, it will be very expensive to conduct a full census, in case a need for local-level data arises for policy purposes. In this setting, our estimates provide the next best alternative and a cost-effective method for obtaining population density that policy makers may use.

Our results make the case for national statistics offices to apply this method using open-source geo-spatial data and administrative area measures in combination with frequently conducted surveys to produce accurate local population estimates in the between-census years and in areas where surveys are not conducted.

For a survey to be utilized for the purposes of our model, there are three main requirements. First, the survey should be nationally representative so that it can be utilized to provide estimates across the country. This is not a stringent requirement as nationally representative surveys such as the Demographic Health Surveys (DHS), Labor Force Surveys (LFS), and Living Standards Measurement Surveys (LSMS) are conducted quite frequently. Importantly, these surveys are conducted at a reasonable frequency even in low-income and conflict-ridden countries. For example, the Afghanistan Living Conditions Survey (ALCS) is conducted in portions of Afghanistan (it doesn’t cover conflict areas) every few years; the DHS have been conducted in countries such as Democratic Republic of Congo and Sierra Leone, and the LSMS have been conducted in countries such as Ethiopia and Niger reasonably regularly. Second, the survey must conduct a full census of selected enumeration areas. This is also quite standard. Third, local-level administrative shape files are required to be able to use satellite imagery-based indicators (open-source or commercial), and the surveys should have the local administrative region identifiers or geocoded household location. Increasingly nowadays, shape files at lower administrative levels are being shared by census offices of countries. With established trust and some negotiations, it is feasible to obtain local administrative level identifiers from survey and statistical offices.

The Sri Lankan Department of Census and Statistics conducts the HIES every three years; the most recent one was fielded in 2019, and the earlier surveys this past decade were in 2012-13, and in 2016. Furthermore, Sri Lanka runs a continual Labor Force Survey that could also be used for this purpose. Therefore, the results support the case for the Department of Census and Statistics to keep careful track of the number of households identified in the relisting phase, and use that information to generate revised local population estimates.

While we presented above a spatial model, without a time-dimension, the changes of population density over time and space can also be estimated simultaneously. There are two potential methods to do this. The first would be to estimate a spatial-temporal model that explicitly accounts for correlation across space and time. Adding earlier periods of data to the model would generate more precise estimates, even after accounting for inter-temporal correlation, but could lead to less accurate predictions if the relationship between predictors and population density changes over time. The second approach would be to estimate a dynamic panel data model that conditions on past population density, measured for example in the census. For the latter to work, the imagery-based indicators would have to predict changes in population density over time. It is an open question of whether they can do so, and this an interesting area for further research.

## Supporting information

S1 File (ZIP)

S1 Appendix Estimating GN (village)-population from the HIES. [29].
(PDF)

S2 Appendix The 55 divisional secretariat divisions (sub-districts). (PDF)

S1 Table Marginal effects of LASSO-selected variables from the full model in Table 4. (PDF)

S2 Table Comparison with random forest models. [13].
(PDF)

S3 Table Accuracy of population density estimates, by training sample size. (PDF)

S4 Table Out-of-sample accuracy of HIES-based village population count estimates, 55 sub-districts. (PDF)

S5 Table Open-sourcely available population products. [28, 30–33].
(PDF)
