# Supplementary material for: Estimating small-area population density in Sri Lanka using surveys and Geo-spatial data
Source: PLoS One. 2020 Aug 5;15(8):e0237063. doi: 10.1371/journal.pone.0237063 (PMC7406065; doi:10.1371/journal.pone.0237063)
Supplement: S1 Appendix — [29]. (PDF) [file pone.0237063.s002.pdf]

S1 Appendix. Estimating GN (village)-population from the HIES

To estimate village-level population density from the Sri Lankan Household Income and Expenditure Survey (HIES), we use a special version of the HIES obtained from the Department of Census and Statistics (DCS) that contains village identifiers. We infer the village population from sample weights using the following identity:

$$W_{h,v} = \frac{1}{prob(h|PSU)} \frac{1}{prob(PSU)}, \tag{4}$$

where $W_{h,v}$ represents the sample weight assigned to household h in a particular village v. Equation 4 reflects the standard definition of the sample weight which equals the inverse probability of a household being selected. Because of the two-stage sampling design employed by the DCS, the probability of the household being surveyed is equal to the product of the probability of the Primary Sampling Unit (PSU) being selected ($prob(PSU)$) and the probability that the household is selected conditional on the PSU being selected ($prob(h|PSU)$). The probability that the household is selected conditional on its PSU being selected is:

$$prob(h|PSU) = \frac{HU_{sample}}{HU_{psu}} \tag{5}$$

HU_{sample} indicates the number of household units in the sample, and HU_{psu} indicates the number of household units in the PSU. Equation 5 indicates that each housing unit within a PSU has an equal probability of selection. The numerator can easily be calculated from the HIES sample, while the denominator needs to be backed out from the sample weights. An important note is that the denominator is updated each time a new survey is conducted by survey teams that lists *all* households in sampled PSUs.

The second term of equation 4 is the inverse probability that the PSU is selected. The probability that the PSU is selected is equal to the share of census housing units in that PSU [29]. To estimate population at the village level, we utilize the following application of Bayes' rule:

$$Prob(PSU) = Prob(PSU|v) * Prob(v) \tag{6}$$

This decomposition is a mathematical identity rather than a description of the actual sample design. It is useful because the first term in equation 6 can be rewritten as follows:

$$Prob(PSU|v) = \frac{HU_{psu}}{HU_v} \tag{7}$$

This indicates that, if hypothetically the village of the selected PSU was known, the probability that the PSU was selected for the sample is the share of that village's housing units contained in that PSU. This identity holds only for villages that exactly contain one PSU, which applies to 97 percent of the villages in the HIES sample. 93.5% of the PSUs in the sample are located in single PSU-villages. Substituting equations 5, 6, and 7 in 4 gives:

$$W_{h,v} = \frac{HU_v}{HU_{sample}} * \frac{1}{prob(v)} \tag{8}$$

$$HU_v = W_{h,v} * HU_{sample,v} * Prob(v) \tag{9}$$

The first term on the right-hand side of equation 9 is the sample weight for household h in a particular village. The second is the number of households in that village that were sampled in the survey. The final term is the probability that the village is in the sample, which needs to be estimated using open-source indicators on the population of each village in the 2011 census. The village population estimates are available at LankaStatMap (http://www.map.statistics.gov.lk/). We merge census population with the HIES data at the village level and estimate the probability of village selection as the following:

$$Prob(v) = 1 - \prod_{i \neq v} \frac{HU_v}{HU_{country} - \sum_{j=1}^{i} HU_j} \tag{10}$$

The probability that a village was selected for the sample is equal to one minus the probability that the village was not selected. The probability that a village was not selected is equal to the product, across all other villages in the sample, of that village not being selected each time a PSU is drawn for the sample. Since the sample is without replacement, in each draw the probability that a village is not selected is equal to one minus the share of remaining non-sampled housing units in each round. We simulate this probability using the actual other villages selected for the sample. This can be calculated for each village based on open-source village population data.

Finally, we multiply the inferred number of housing units in the village from equation 9 by the average household size of that village in the sample, to obtain an estimate of the village-level population. This is then divided by the physical size of the village, derived from the boundary file, to obtain an estimate of the population density of each village in the sample.

This procedure generates estimates that correspond reasonably closely to the census. However, accuracy could be improved by obtaining direct counts of the number of housing units in each sample PSU, rather than indirectly obtaining estimates from the sample weights. This would eliminate sources of approximation error, such as dropping three percent of the villages that contain multiple PSUs. Therefore, the estimation accuracy using the HIES-based density measure can be interpreted as a lower bound. Because these approximation errors are largely uncorrelated with the satellite-based independent variables in the model, using the HIES-based density approximation as the dependent variable only slightly decreases the accuracy of the estimates reported in table 5. This suggests that household survey weights, under the proper conditions, can be combined with readily available census-based population counts to obtain reasonable estimates of population density at fine geographic levels in surveyed areas.
