# Supplementary material for: Estimating small-area population density in Sri Lanka using surveys and Geo-spatial data
Source: PLoS One. 2020 Aug 5;15(8):e0237063. doi: 10.1371/journal.pone.0237063 (PMC7406065; doi:10.1371/journal.pone.0237063)
Supplement: S2 Appendix — (PDF) [file pone.0237063.s003.pdf]

S2 Appendix. The 55 Divisional Secretariat divisions (sub-districts) Ambagamuwa, Ambalantota, Ambanpola, Bandaragama, Biyagama, Bulathsinhala, Colombo, Dehiwala, Devinuwara, Dodangoda, Doluwa, Dompe, Galle Four Gravets, Hali Ela, Hambantota, Homagama, Horana, Ingiriya, Kaduwela, Kalutara, Kamburupitiya, Katana, Kattankudy, Kelaniya, Kesbewa, Kirinda Puhulwella, Kolonnawa, Kotapola, Kotmale, Kurunegala, Madurawala, Maharagama, Malimbada, Manmunai North, Matara Four Gravets, Moratuwa, Nagoda, Negombo, Nuwara Eliya, Nuwaragam Palatha East, Padukka, Panadura, Panwila, Puttalam, Ratmalana, Rattota, Seethawaka, Sri Jayawardanapura Kotte, Thihagoda, Thimbirigasyaya, Tissamaharama, Udapalatha, Udunuwara, Ukuwela, and Uva-Paranagama.
