# Supplementary material for: Estimating small-area population density in Sri Lanka using surveys and Geo-spatial data
Source: PLoS One. 2020 Aug 5;15(8):e0237063. doi: 10.1371/journal.pone.0237063 (PMC7406065; doi:10.1371/journal.pone.0237063)
Supplement: S1 Table — (PDF) [file pone.0237063.s004.pdf]

S1 Table. Marginal Effects of LASSO-Selected Variables from the full model in table 4

	National Sample (1)	55-sub district sample (2)
Open-source indicators		
Night-time lights (March 2014), Maximum	3.006***	0.654
	(1.142)	(1.514)
Mean Slope		-67.73***
		(16.57)
Built-up area from Global Urban Footprint (GUF)	5.749***	
	(2.228)	
Built-up area from Global Human Settlement Layer (GHSL)	4.362***	
	(1.177)	
Built-up area from Facebook	12.22***	-3.281
	(2.657)	(5.707)
Built-up area from GUF+	7.231***	25.67***
	(1.526)	(3.756)
Commercially-procured indicators		
% of roads that are paved city (4 m width)		8.884***
		(2.914)
% of roads that are main paved (5 m width)		-9.340***
		(3.62)
% of roads that are minor paved (4 m width)		-0.422
		(4.537)
% shadow pixels covering valid area		50.06***
		(16.31)
Normalized Difference Vegetation Index (NDVI) (mean) scale 32		-964.1**
		(457.4)
Total built-up area		0.00406***
		(0.00117)
Fraction of total roofs that are clay		-35.95***
		(4.737)
Fraction of total roofs that are aluminum		-22.79***
		(8.555)
Fraction of total roofs are asbestos		-27.37***
		(7.247)
log number of cars		-251.5***
		(75.94)
% of Village agriculture that is paddy		3.907***
		(1.338)
Pantex (human settlements) mean, scale 8m		-173.9
		(283.6)
Histogram of Oriented Gradients (scale 16m), mean		-17.14**
		(8.38)
Gabor filter (scale 64m, features 6), mean		-387.3
		(1,179)
Gabor filter (scale 64m, features 14), mean		1,927
		(1,409)
Geographical descriptors		
Ln Village area	-1,633***	-2,263***
	(82.59)	(264.2)
Binary for urban Villages	161.0**	
	(73.13)	
Observations	12,088	1,051

Note: Marginal effects from the Poisson regression model of census population density on variables selected from LASSO selection are reported. Marginal effects of district indicators are not reported, but are available upon request. Standard errors clustered at the sub-district level in parentheses; *** implies $p < 0.01$, ** implies $p < 0.05$, and * implies $p < 0.1$.
