# Supplementary material for: Estimating small-area population density in Sri Lanka using surveys and Geo-spatial data
Source: PLoS One. 2020 Aug 5;15(8):e0237063. doi: 10.1371/journal.pone.0237063 (PMC7406065; doi:10.1371/journal.pone.0237063)
Supplement: S2 Table — [13]. (PDF) [file pone.0237063.s005.pdf]

S2 Table. Comparison with Random Forest Models

Geo-spatial Indicators				Random Forest			LASSO		
Low-res (open)	High-res (open)	Texture (comm.)	Object (comm.)	Out-of-bag R^2	RMSE	MAE	Out-of-sample R^2	RMSE	MAE
A. All sectors									
i. National sample									
×				0.767	1282	477	0.702	1463	563
	×			0.783	1237	390	0.750	1323	459
ii. 55-sub districts									
×				0.677	2088	948	0.677	1948	928
	×			0.707	1984	784	0.710	1927	818
		×		0.719	1942	779	0.755	1845	808
			×	0.792	1673	684	0.830	1506	698
B. Rural/Estate									
iii. National sample									
×				0.776	696	329	0.726	788	375
	×			0.832	601	246	0.808	653	289
iv. 55-sub districts									
×				0.807	968	510	0.754	990	550
	×			0.847	855	383	0.838	848	424
		×		0.849	850	380	0.882	788	415
			×	0.872	781	352	0.869	747	355
C. Urban									
v. National sample (urban)									
×				0.598	3966	2285	0.540	4105	2344
	×			0.612	3890	2079	0.569	3999	2343
vi. 55-sub districts (urban)									
×				0.424	3680	2084	0.511	3277	1917
	×			0.461	3559	1888	0.525	3357	1909
		×		0.491	3457	1850	0.668	2897	1676
			×	0.626	2984	1620	0.804	2226	1446

Note: Open refers to Open-source imagery, and comm. refers to commercially-procured imagery. The models use the census village population density as the dependent variable. The following independent variables were used in all models: Different types of satellite imagery sources as described in panel B in table 1, district fixed effects, a binary indicator for urban villages, and log village-area. "Low-resolution (open)" indicators refer to the low-resolution and Open-source indicators as defined in table 1 in the main text. "High-res (open)" indicators refers to all open-source based indicators, including the high-resolution imagery based indicators described in table 1. "Texture (comm.)" indicators include very high-resolution texture features based on Digital Globe from [13] as defined in table 1. "Object (comm.)" imagery refers to all open-source indicators, and commercially-procured texture indicators, and object identifiers, such as cars, roofs etc., as defined as in table 1. The out-of-sample R^2, Mean Absolute Error (MAE), and Root Mean Squared Error (RMSE) for the LASSO based Poisson regression are from tables 8-10.
