# Supplementary material for: Estimating small-area population density in Sri Lanka using surveys and Geo-spatial data
Source: PLoS One. 2020 Aug 5;15(8):e0237063. doi: 10.1371/journal.pone.0237063 (PMC7406065; doi:10.1371/journal.pone.0237063)
Supplement: S3 Table — (PDF) [file pone.0237063.s006.pdf]

S3 Table. Accuracy of Population Density Estimates, by Training Sample Size

		In-sample	Out-of-sample		
Sample Type	Number of Villages	Pseudo R^2	Pseudo R^2	MAE	RMSE
Medium sample	1178	0.918	0.842	1490	712
Small sample (50% of medium)	593	0.908	0.843	1447	698
Very small sample (50% of small)	292	0.920	0.832	1691	738

Note: The results are based on a Poisson regression model whose dependent variable is census village population density, and the independent variables are selected based on LASSO regularization among the following: All open-source and commercially-procured imagery sources as described in panel B in Table 1, sectoral and district fixed effects, and log village-area. The out-of sample R^2 is obtained from stata's crossfold command using five-fold cross-validation. MAE refers to mean absolute error and RMSE refers to Root Mean Squared Error.
