# Supplementary material for: Estimating small-area population density in Sri Lanka using surveys and Geo-spatial data
Source: PLoS One. 2020 Aug 5;15(8):e0237063. doi: 10.1371/journal.pone.0237063 (PMC7406065; doi:10.1371/journal.pone.0237063)
Supplement: S4 Table — (PDF) [file pone.0237063.s007.pdf]

S4 Table. Out-of-sample Accuracy of HIES-based Village Population Count Estimates, 55 Sub-Districts

	R^2	SRC	Mean AE	Median RE	Median RE
A.Model estimates, Open-source & Commercially-procured satellite imagery					
HIES-based	0.26	0.51	642	42%	30%
HIES-based (with balance correction)	0.24	0.49	641	42%	31%
Census-based	0.28	0.36	694	58%	32%
B.Model estimates, Open-source satellite imagery					
HIES-based	0.23	0.33	668	42%	30%
HIES-based (with balance correction)	0.23	0.34	666	42%	30%
Census-based	0.25	0.28	792	57%	31%

Note: Note: HIES=the Household Income and Expenditure Survey; GPW=Gridded Population of the World; GHSL=Global Human Settlement Layer; GUF= Global Urban Footprint; AE= Absolute; RE=Relative Error; SRC=Spearman Rank Correlation. We retain only the 1,178 villages in the 55 sub-districts for which the commercially-available data were obtained. The model is estimated using population count as the dependent variable on sub-sample of 414 villages covered in 2012-2013 HIES survey. All models include district fixed effects, urban-area dummy, and log village area. Out-of-sample estimates for testing the accuracy with the census are obtained on 946 villages not covered in the HIES. The weights for the HIES-based model are $\frac{1}{Pop_v}$. The balanced corrected weights are $\frac{1}{(PopXI\hat{N}_v)}$.
