# Supplementary material for: Estimating small-area population density in Sri Lanka using surveys and Geo-spatial data
Source: PLoS One. 2020 Aug 5;15(8):e0237063. doi: 10.1371/journal.pone.0237063 (PMC7406065; doi:10.1371/journal.pone.0237063)
Supplement: S5 Table — [28, 30–33]. (PDF) [file pone.0237063.s008.pdf]

S5 Table. Open-sourcely Available Population Products

Data Source	Resolution	Census information and other details	Distribution technique
LandScan [28]	30 arc seconds (approximately 926m)	LandScan uses annual mid-year national population estimates from the Geographic Studies Branch, US Bureau of Census (not the official census of the country).	LandScan distributes census counts in an area across grids based on the likelihood of being populated. This likelihood coefficient is calculated based on geospatial characteristics, including, roads, elevation, slope, and light intensity.
Facebook's High-Resolution Settlement Layer (HRSL) [30]	1 arc-second (approximately 30m)	Most recent censuses. The population data have been developed for 18 countries: Algeria, Burkina Faso, Cambodia, Ghana, Haiti, Ivory Coast, Kenya, Madagascar, Malawi, Mexico, Mozambique, the Philippines, Puerto Rico, Rwanda, South Africa, Sri Lanka, Tanzania, Thailand, and Uganda.	High-resolution (0.5m) satellite imagery from Maxar (formally Digital-Globe). The satellite imagery were classified as settled or not based on computer vision techniques. Proportional allocation was used to distribute population data from sub-national census data based on the settlement extents.
WorldPop 2010 and 2015 [31]	100 m	WorldPop 2015 uses the 2010 round of censuses; WorldPop 2010 uses the 2000 round of censuses	A semi-automated dasymetric modelling approach using random forest method incorporating geospatial datasets (e.g. settlement locations, settlement extents, land cover, roads, building maps, health facility locations, satellite nightlights, vegetation, topography, refugee camps).
Gridded Population of the World (GPW) v3 (2010) and v4 (2015) [32]	GPW v4: 30 arc second (approximately 1 km); GPW v3: 2.5 arc minute (about 5 km)	V4 uses the 2010 round of censuses, and V3 uses the 2000 round of censuses	Uniform weighting method at the GN (village) administrative level
Global Human Settlement (GHS) [33]	250m	Residential population estimates from CIESIN GPWv4	Population estimates are disaggregated from census or administrative units to grid cells, informed by the Global Human Settlement Layer (GHSL) built-up area measures.

637
